# Mapping the Altered Landscape of TCR Repertoire in Autoimmune Diseases

**DOI:** 10.1111/imr.70174

**Published:** 2026-09-04

**Authors:** Celine Albalaa, Martin Pezous, David Klatzmann, Encarnita Mariotti‐Ferrandiz

**Affiliations:** ^1^ Sorbonne Université, INSERM, Immunoregulation‐Immunopathology‐Immunotherapy (i3) Paris France; ^2^ Institut Universitaire de France (IUF) Paris France

## Abstract

Autoimmune diseases arise from the breakdown of immune tolerance through complex interactions between genetic predisposition, environmental exposures, and adaptive immune responses. High‐throughput T‐cell receptor (TCR) repertoire sequencing has transformed our ability to characterize these responses, providing unprecedented insights into clonal dynamics, antigen‐driven selection, and immune history. In this review, we summarize the major alterations of TCR repertoires reported across autoimmune diseases, including changes in diversity, clonal expansion, repertoire architecture, and tissue distribution. We discuss the principal biological and technical challenges that currently limit repertoire interpretation, with particular emphasis on the concept that bulk repertoires represent composite mixtures of biologically distinct T‐cell populations. Finally, we highlight emerging approaches integrating single‐cell profiling, computational modeling, and antigen‐specificity inference that are reshaping the field. We propose that TCR repertoires should be viewed as dynamic molecular footprints of autoimmune disease, providing a systems‐level framework to improve mechanistic understanding, biomarker discovery, and the development of precision immunotherapies.

## 1 A Brief Overview of T‐Cells in Autoimmune Diseases

### 1.1 Autoimmune Diseases: A Continuum of Adaptive Immune Dysregulation

Autoimmune diseases (ADs) are chronic and debilitating diseases representing the second cause of chronic illness worldwide, with a critical societal burden due to the high costs of poorly effective treatments and life‐threatening side effects negatively impacting a patient's quality of life. None of the available treatments are disease‐specific or curative, resulting in an unmet need for novel therapeutics and better understanding of pathophysiology [1, 2]. More than 80 ADs have been characterized so far and their incidence and prevalence have been increasing over the last decades worldwide [3, 4, 5], with an imbalance in women/men ratio [6, 7]. ADs are usually classified either as systemic ADs, with systemic tissue damage (such as systemic lupus erythematosus (SLE), rheumatoid arthritis (RA), Sjögren disease (SjD)) or as organ‐specific AD (such as type‐1‐diabetes (T1D), multiple sclerosis (MS)), altogether forming a heterogeneous group of disorders rather than purely distinct diseases [8]. It is generally accepted that ADs result from a patient's T and B lymphocyte adaptive immune response attacking their own body. Many studies have evidenced the important role of innate immunity in several ADs, notably in the disease onset, yet, the innate cells greatly contribute to the activation of autoreactive T‐ and B‐lymphocytes [9]. Moreover, various genetic variant associations linked to T‐ and B‐cell biology have been identified, including HLA alleles, T‐cell activation or autoreactive B‐cell differentiation related genes [10, 11, 12, 13, 14, 15, 16, 17], some of which are shared between various ADs. Environmental factors, such as chemicals, tobacco exposure, drugs's intake, endocrine disruptors, infections and commensal microorganisms also impact on the incidence and prevalence of the diseases [5, 16, 18, 19, 20, 21]. Finally, individuals with one AD may develop other ADs over time, some disease combinations being common, such as SLE, RA and SjS [22, 23]. These three ADs not only frequently overlap but also share clinical serological and immunological characteristics suggesting potential common aetiological factors and pathophysiology [24, 25], supporting the continuum of diseases.

Diagnosis of ADs is mainly based on clinical evaluation and standard laboratory measurements, including autoantibodies, the product of autoreactive B‐cells. However, the biological relevance of these biomarkers remains poorly understood for some diseases.

### 1.2 Mapping the Adaptive Immune Landscape of Autoimmunity

T‐ and B‐cells play a complex role in ADs. These roles are supported by recent successful therapies targeting either T‐cells (T‐cell depletion using anti‐CD3 immunotherapy in T1D [26]) or B‐cells (B‐cell depletion therapies such as anti‐CD20 in various ADs [27]) or B‐T cell crosstalk (anti‐CD40 in pSjD [28]) further support the major role of these cells in AD. Nonetheless, such pan‐B and pan‐T cell depletion therapies target all the adaptive immune cells, including those cells required for the organism's protection against pathogens, limiting the generalization of such therapies. B‐cell markers are insufficient to understand and delineate ADs; T‐cells and their crosstalk with B‐cells remain unexplored in the context of ADs.

In many autoimmune diseases, B cells play a central role through the production of pathogenic autoantibodies. This is particularly evident in SLE, where anti‐nuclear autoantibodies (ANA) drive immune‐complex formation and tissue injury [16, 29, 30], but also in myasthenia gravis, pemphigus vulgaris, autoimmune thyroid diseases, and ANCA‐associated vasculitis, where autoantibodies directly contribute to disease pathology [31, 32, 33]. In RA, anti‐citrullinated protein antibodies (ACPA) are key factors and biomarkers. Their production is supposedly B‐ and T‐cell dependent, suggesting possible anti‐citrullinated protein‐specific T cells [34, 35]. In T1D, autoantibodies targeting insulin, GAD65, IA‐2, or ZnT8 are among the earliest detectable signs of autoimmunity and represent powerful predictors of disease progression, although β‐cell destruction is thought to be primarily mediated by autoreactive T cells [36, 37].

Accumulating evidence indicates that the contribution of B cells extends beyond humoral immunity. Through antigen presentation, cytokine production, and reciprocal interactions with T cells, B cells participate in the initiation and maintenance of autoimmune responses across a broad spectrum of diseases, including RA, MS, T1D, and pSjD [16, 38, 39]. As such, B‐cell targeting therapies have become a cornerstone of treatment for several autoimmune diseases, demonstrating that B cells contribute to pathogenesis not only through autoantibody production but also through antigen presentation, cytokine secretion, and the maintenance of pathogenic T‐cell responses [16, 40]. Incomplete depletion of tissue‐resident B cells and the persistence of long‐lived plasma cells often limit the depth and durability of clinical responses. Building on these observations, next‐generation immune‐targeting approaches, including CD19‐directed CAR‐T cells, have induced prolonged drug‐free remissions in severe refractory systemic lupus erythematosus [16], while emerging T‐cell engager strategies may offer similarly potent and potentially more scalable approaches to eliminate pathogenic B‐cell populations and achieve durable immune resetting [41]. As such, autoimmune diseases emerge not from isolated B‐cell abnormalities but from dysregulated adaptive immune networks sustained by reciprocal T‐B‐cell interactions. Whether antigen‐specific T cells may orchestrate many of these interactions by providing help to autoreactive B cells and directing tissue‐specific immune responses has become central to deciphering autoimmune disease mechanisms.

As such, T cells are also central in the initiation, propagation, and maintenance of autoimmune diseases. CD4 T helper subsets orchestrate inflammatory responses through cytokine production and immune‐cell recruitment, with Th1 and Th17 cells playing prominent roles in diseases such as MS, RA, psoriasis, inflammatory bowel disease (IBD), and T1D [42, 43]. Autoreactive T cells may contribute to disease through multiple, non‐mutually exclusive mechanisms. T‐cells, and notably CD4 T‐cells, are associated with synovial inflammation in early and established RA [44], with clonal T‐cell expansion observed in the early stages [45]. In parallel, follicular helper T cells (Tfh) have emerged as critical mediators of humoral autoimmunity. Through IL‐21 production and direct interactions with B cells, Tfh cells promote germinal‐center reactions, affinity maturation, and autoantibody production. Expanded Tfh populations have been reported in SLE, RA, ANCA‐associated vasculitis, pSjD, and other antibody‐mediated autoimmune diseases, where they correlate with disease activity and autoantibody levels [46, 47].

Conversely, defects in regulatory T cells (Tregs), whether quantitative, functional, or tissue‐specific, contribute to the breakdown of peripheral tolerance and have been reported across numerous autoimmune conditions [48, 49]. The importance of Treg‐mediated regulation is further supported by the development of therapeutic approaches aimed at restoring Treg homeostasis, including low‐dose IL‐2 administration and adoptive Treg therapies in diseases such as SLE and T1D [16, 50, 51, 52, 53, 54].

Beyond their helper functions, T cells can also act as direct mediators of tissue injury. CD8 cytotoxic T cells are recognized as major pathogenic effectors in several autoimmune diseases, particularly in organ‐specific disorders. In T1D, autoreactive CD8 T cells directly mediate pancreatic β‐cell destruction and constitute dominant immune populations within islets [26, 55, 56, 57]. Interestingly, their presence in the blood does not signify disease as they are also found in healthy volunteers [16]. Similarly, clonally expanded CD8 T cells are enriched within inflammatory lesions in MS, where they are thought to contribute to neuronal and oligodendrocyte injury [58, 59]. Tissue‐resident and clonally expanded CD8 T‐cell populations have also been implicated in a broad range of autoimmune diseases, including rheumatoid arthritis, primary Sjögren's syndrome, vitiligo, ankylosing spondylitis, and lupus nephritis. Recent single‐cell and paired TCR studies have revealed that these populations frequently display hallmarks of chronic antigen stimulation, tissue adaptation, and local clonal expansion, supporting a central role for persistent antigen‐driven responses in autoimmune pathology [60, 61, 62]. CD8 T‐cells may also play a role in the disease through pro‐inflammatory cytokine release [63], yet a recent study has suggested their antigen‐independent role in the maintenance of the pro‐inflammatory environment [64]. Among autoimmune diseases, HLA‐B27‐associated ankylosing spondylitis (AS) and related axial spondyloarthritis (axSpA) provide one of the most compelling examples linking TCR repertoire alterations to disease pathogenesis. Convergent and shared CD8^+^ TCRs have been identified across patients and enriched at sites of inflammation [65, 66, 67], with subsequent studies linking these receptors to HLA‐B27‐restricted antigen recognition and microbial–self cross‐reactivity [68, 69] and providing initial proof of concept for therapeutic targeting of repertoire‐defined T‐cell populations [70]. These findings illustrate how deep characterization of disease‐associated TCR repertoires can move beyond documenting T‐cell involvement to reveal antigen‐driven mechanisms and potentially identify selectively targetable T‐cell populations.

Altogether, B cells and T cells do not act in isolation. Increasing evidence indicates that autoimmune diseases emerge from complex interactions between T cells, B cells, innate immune populations and target tissues as well as the environment. In particular, T‐cell–B‐cell crosstalk has emerged as a major pathogenic axis across many disorders. Tfh‐driven B‐cell activation promotes the generation of autoreactive clones and pathogenic autoantibodies, while B cells in turn sustain T‐cell activation through antigen presentation and cytokine production. Altogether, these observations place antigen‐specific T‐cell responses at the center of autoimmune pathogenesis and provide a strong rationale for investigating T‐cell receptor (TCR) repertoires to identify disease‐associated clones, characterize immune dysregulation and uncover shared or disease‐specific mechanisms of autoimmunity.

### 1.3 Antigen Specificity: The Hidden Topography of Autoimmune Diseases

Although antigen specificity is the defining property of adaptive immunity, our understanding of the antigens and antigen‐specific lymphocyte responses driving autoimmune diseases remains remarkably incomplete. In a limited number of diseases, candidate autoantigens have been identified and extensively characterized. T1D represents one of the best‐studied examples, with autoreactive T‐cell responses directed against insulin, GAD65, IA‐2, ZnT8, and other β‐cell antigens being detected years before clinical onset [16, 21, 55, 71, 72, 73]. Nevertheless, the limited success of most antigen‐specific therapeutic approaches highlights the complexity of autoimmune responses and suggests that disease pathogenesis cannot be fully explained by a small set of dominant antigens [74]. This is also exemplified with the very recently evaluated Diamyd vaccine, based on the human recombinant GAD65, which showed clinical benefit only in a subset of HLA‐DR3‐DQ2‐positive T1D patients [75]. Similarly, while autoantibodies against nuclear antigens in SLE, ACPA in RA, or Ro/La antigens in primary SjD constitute important diagnostic and prognostic markers [76, 77], the corresponding T‐cell specificities remain only partially understood. Adding further complexity, antigen specificity is unlikely to remain static throughout disease evolution. Tissue inflammation and destruction continuously release new self‐antigens, creating opportunities for the recruitment and expansion of lymphocytes recognizing previously untargeted epitopes, a process referred to as epitope spreading. Such diversification of immune responses has been documented in several autoimmune diseases and is thought to contribute to disease progression, organ dissemination, and therapeutic resistance. In parallel, molecular mimicry between microbial and self‐antigens may initiate or amplify autoreactive responses, further blurring the distinction between environmental and self‐derived triggers [78, 79]. Together, these observations suggest that autoimmune diseases are not characterized by fixed antigenic targets but rather by dynamic and evolving antigen‐specific immune landscapes.

The incomplete understanding of antigen specificity represents a major obstacle to both mechanistic insight and therapeutic development. Because TCR constitutes a molecular record of antigen‐driven immune responses, characterization of TCR repertoires offers a unique opportunity to investigate the antigenic architecture of autoimmune diseases. Beyond the identification of disease‐associated clonotypes, repertoire analyses can provide insights into clonal expansion, immune selection, convergent responses, disease heterogeneity, and longitudinal immune evolution, thereby offering an unprecedented window into the mechanisms that sustain autoimmunity.

## 2 Biological Foundations of the TCR Repertoire

**FIGURE 1:**
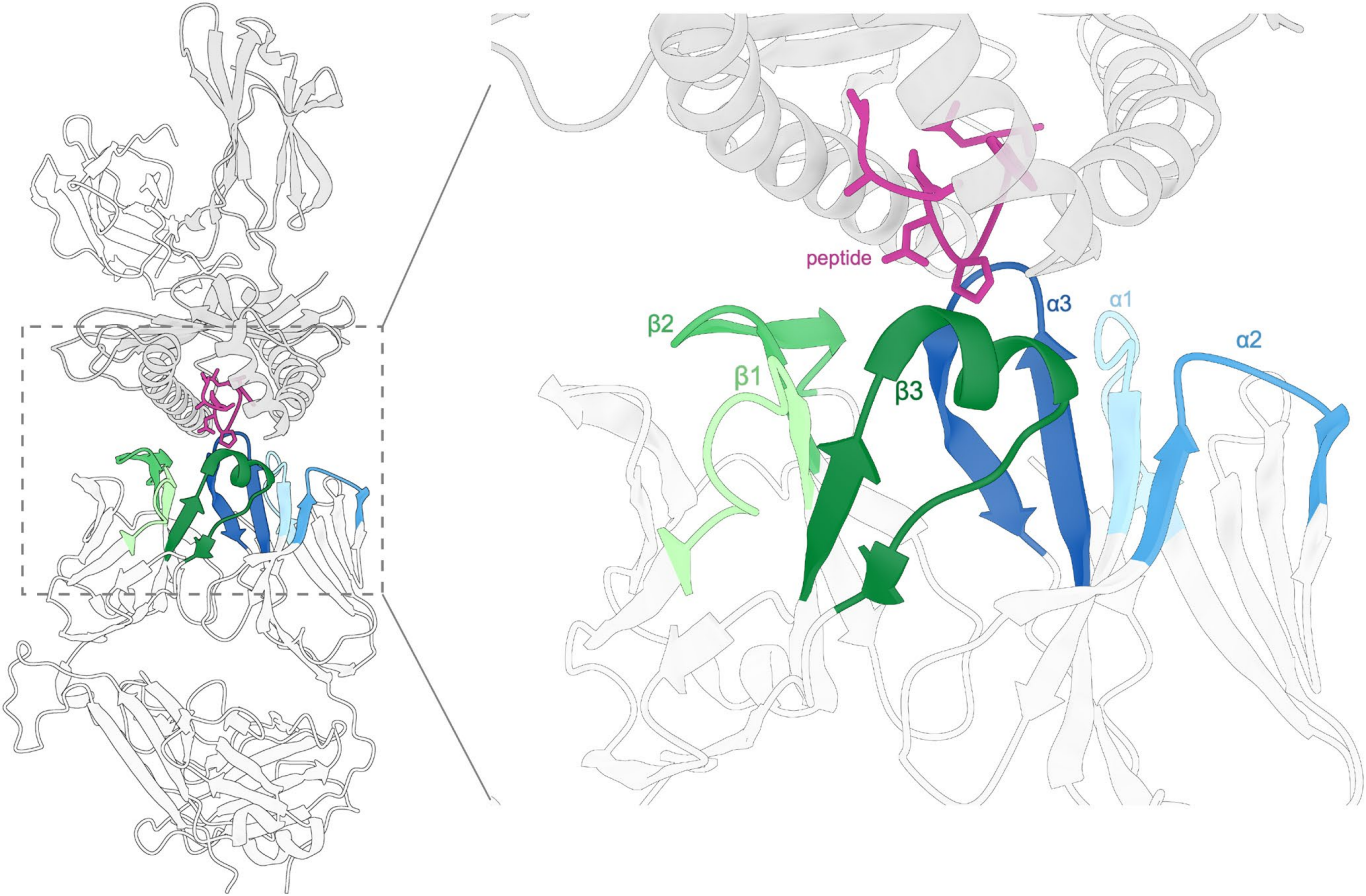
Structure of the TCR–peptide–MHC interface using the 1E6 TCR bound to HLA‐A*02:01 presenting the preproinsulin peptide ALWGPDPAAA (PDB: 3UTT). The left panel shows the full TCR–pMHC complex, with the dashed box indicating the interface region enlarged in the right panel. The peptide is shown as sticks and colored purple, TCRα CDR loops are shown in shades of blue, and TCRβ CDR loops are shown in shades of green; the remaining TCR and MHC scaffold is shown as a gray cartoon. CDR loop boundaries were annotated using TCR3d, and molecular graphics were rendered with UCSF ChimeraX.

T cells mediate antigen‐specific immunity through their TCR, a heterodimeric transmembrane protein complex composed of an α (TRA) and β (TRB) chain in approximately 95% of T cells. The TCR repertoire is generated during thymocyte development through V(D)J recombination [80, 81], a stochastic somatic process in which variable (V), diversity (D), and joining (J) gene segments are randomly assembled, with the β chain undergoing V‐D‐J recombination and the α chain V‐J recombination only, yielding a theoretical diversity estimated from 10^15^ [16] to 10^61^ distinct sequences [82]. The complementarity‐determining region 3 (CDR3), often considered the primary determinant of antigen specificity [83, 84], is particularly diverse because it spans the recombination junction and is further shaped by nucleotide insertions and deletions at junctions [85]. While CDR1 and CDR2, encoded within the germline V segments, primarily contact the conserved helical residues of the MHC molecule, the highly variable CDR3 loop principally engages the bound peptide [86, 87, 88]. TCRs recognize these peptide‐MHC complexes on the surface of antigen‐presenting cells (APCs), as shown in Figure 1, including dendritic cells, macrophages, and B cells, which internalize, proteolytically process, and present antigenic peptides to circulating T cells [89]. This recognition is MHC class‐restricted: CD8^+^ cytotoxic T cells engage peptides on MHC Class I, expressed ubiquitously on nucleated cells, while CD4^+^ T cells engage peptides on MHC Class II, largely restricted to professional APCs [90, 91, 92].

This initially vast diversity is then shaped by sequential thymic selection checkpoints [93]. Positive selection, mediated by cortical thymic epithelial cells presenting unique self‐peptide‐MHC complexes generated in part by thymoproteasomes, rescues thymocytes whose TCRs engage self‐MHC with sufficient affinity [94]. Negative selection subsequently eliminates clones exhibiting high‐affinity self‐reactivity, removing an estimated half to two‐thirds of all positively selected thymocytes [95]. Thymic selection is, however, inherently imperfect: 25%–40% of self‐reactive clones can escape deletion, particularly low‐avidity clones and cells bearing a second TCR that masks autoreactivity [16, 96]. A third thymic outcome, differentiation into Foxp3^+^ regulatory T cells (Tregs), captures autoreactive thymocytes across a broad self‐reactivity window: the TCR‐reactivity threshold required for negative selection is approximately 100‐fold higher than that required for Treg differentiation [97]. This enriches the Treg compartment with cells whose self‐reactivity is strong enough to promote regulatory lineage commitment, but not strong enough to induce clonal deletion [98, 99, 100].

The mature peripheral TCR repertoire retains substantial diversity, with the number of distinct TCRβ clonotypes, defined as the uniquely rearranged TCR sequences, in young adults estimated to range from approximately 10^6^ to 10^8^ [16, 101, 102]. Yet the functional antigen‐recognition capacity of this repertoire extends far beyond its numerical size. This is possible because TCR recognition is highly degenerate: a single TCR can engage many different peptide‐MHC ligands, with estimates suggesting potential cross‐reactivity of more than 10^6^ distinct pMHC complexes [103, 104]. Thus, broad immune surveillance emerges not from a one‐to‐one correspondence between TCRs and antigens, but from a flexible recognition system in which each clonotype samples a large region of the antigenic space.

Peripheral tolerance provides an essential layered backup against autoreactive clones that escape thymic deletion [105, 106, 107]. Tregs, the primary cellular mediators of peripheral tolerance, require antigen‐specific TCR signaling [108, 109] together with IL‐2‐dependent survival and functional support [110, 111, 112], allowing them to suppress autoreactive effector responses through local bystander mechanisms, including inhibitory cytokines, modulation of antigen‐presenting cells, and interleukin 2 (IL‐2) consumption [113, 114, 115, 116]. Critically, gaps in the Treg repertoire can create vulnerabilities in peripheral tolerance: when relevant self‐antigens are absent during thymic development, cognate Tregs may fail to be generated, allowing autoreactive responses against those antigens to emerge [117, 118, 119, 120].

The mature TCR repertoire is therefore not a random collection of clonotypes but a structured and dynamic system whose architecture reflects the cumulative imprint of recombination biases, thymic selection, peripheral regulation, and antigen‐driven expansion. Overall, the TCR repertoire can be understood as an evolving record of developmental history, immune exposure, and functional constraint, whose disruption is increasingly recognized as a central feature of autoimmune pathology.

## 3 T‐Cell Receptors as Molecular Footprints of Autoimmune Responses

**FIGURE 2:**
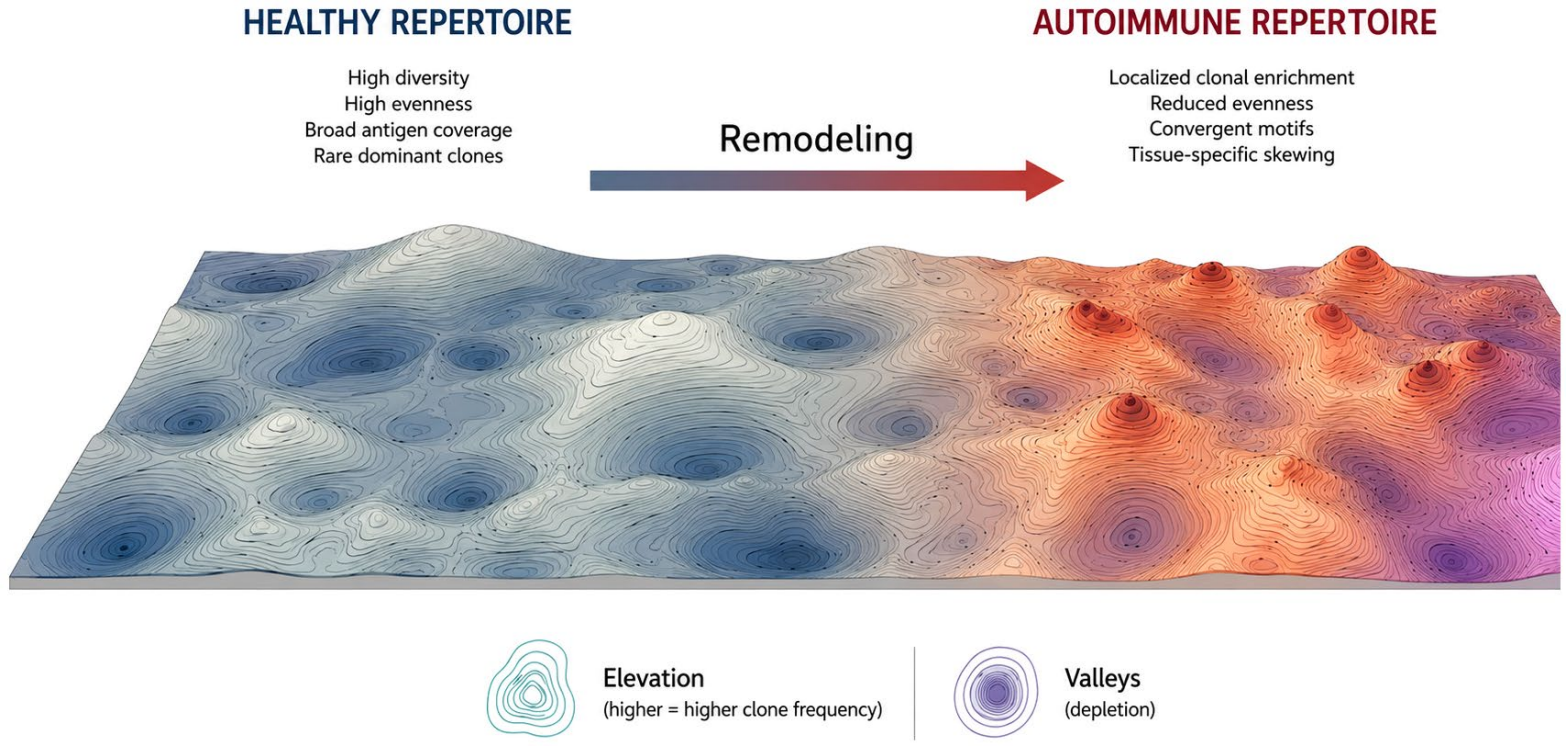
Repertoire remodeling in autoimmune disease. The TCR repertoire is depicted as a terrain‐like antigen‐recognition landscape. Peaks and valleys represent local expansions or depletions of clonotypes associated with specific V/J gene usage, CDR3 length distributions, or biochemical features. The healthy repertoire, shown in blue, displays broad coverage with limited topographic variation. The autoimmune repertoire, shown in red, exhibits more pronounced remodeling, reflecting reduced evenness, localized clonal enrichment, altered coverage, and increased tissue‐specific skewing.

The breakdown of self‐tolerance that characterizes autoimmune disease is not only a functional phenomenon but leaves a distinct imprint on the TCR repertoire itself, as depicted in Figure 2. Advances in high‐throughput sequencing have made it possible to characterize these repertoire alterations with unprecedented resolution [121]. Understanding these features is important not only for elucidating disease mechanisms but also for identifying biomarkers of disease onset, activity, and progression. This section outlines the major TCR repertoire alterations reported across autoimmune diseases, including (i) reduced diversity and oligoclonal expansion; (ii) molecular features of antigen‐driven selection; (iii) altered antigen‐recognition landscapes; (iv) spatio‐temporal and compartmental repertoire dynamics; (v) public clonotypes and repertoire sharing; and (vi) repertoire‐based signatures and their biomarker potential.

### 3.1 Reduced Diversity and Oligoclonal Expansion

Across autoimmune diseases, TCR repertoires frequently show reduced diversity and oligoclonal expansion, reflecting a shift from broadly distributed clonotype usage toward clonal skewing. In SLE, marked repertoire contraction has been observed [122], with longitudinal sequencing reporting a 2.2‐fold reduction in TCR diversity compared with healthy controls (*p* < 0.0002) [123]. In RA, reduced diversity and prominent clonal expansion have been linked to both disease activity [122, 124] and genetic risk background, particularly in memory CD4^+^ T cells; HLA‐DRB1 shared epitope allele‐positive patients showed marked TCRβ repertoire contraction reflected by a 10‐fold increase in clonotype frequency (*p* < 0.001), alongside reduced memory CD4^+^ TCR diversity compared with healthy donors [125]. In Crohn's disease, inflamed mucosal tissue displays decreased diversity, including a reduced D50 diversity index, together with enrichment of expanded clonotypes, in contrast to the broader distribution of rare clonotypes seen in healthy controls [126].

In T1D, however, the picture is more nuanced. One study report significantly reduced peripheral CD4^+^ and CD8^+^ diversity in T1D compared with both T2D patients and nondiabetic controls, accompanied by a higher frequency of expanded clonotypes [127]. Yet other large‐scale analyses have found that repertoire‐level diversity and similarity metrics do not significantly differ between T1D and control groups [128, 129, 130, 131]. This discrepancy likely reflects the subtlety and context‐dependence of T1D‐associated repertoire alterations, including cohort size impact when considering other clinical parameters. Moreover, it also highlights that such repertoire alterations may be undetectable by conventional global diversity metrics and require more sensitive approaches, such as machine learning‐based methods, to resolve.

Collectively, these findings support the notion that autoimmune repertoires frequently undergo clonal narrowing, but reduced diversity is not a universal or uniform signature. Reports of increased TCRβ diversity in some multiple sclerosis cohorts further highlight the context dependence of repertoire remodeling, which varies with disease type, sampled tissue, T‐cell subset, and the analytical metrics applied [132].

### 3.2 Molecular Features of Antigen‐Driven Selection

Beyond changes in overall diversity, autoimmune repertoires often display alterations in clonotype composition, including biased V and J gene usage, preferential V‐J pairing, and shifts in CDR3 length distribution and amino acid composition. These features suggest that autoimmune repertoire remodeling may not simply reflect nonspecific contraction, but could involve selective amplification of receptors with particular structural and sequence properties, potentially consistent with antigen‐driven selection operating within the constraints of thymic selection and MHC restriction.

**FIGURE 3:**
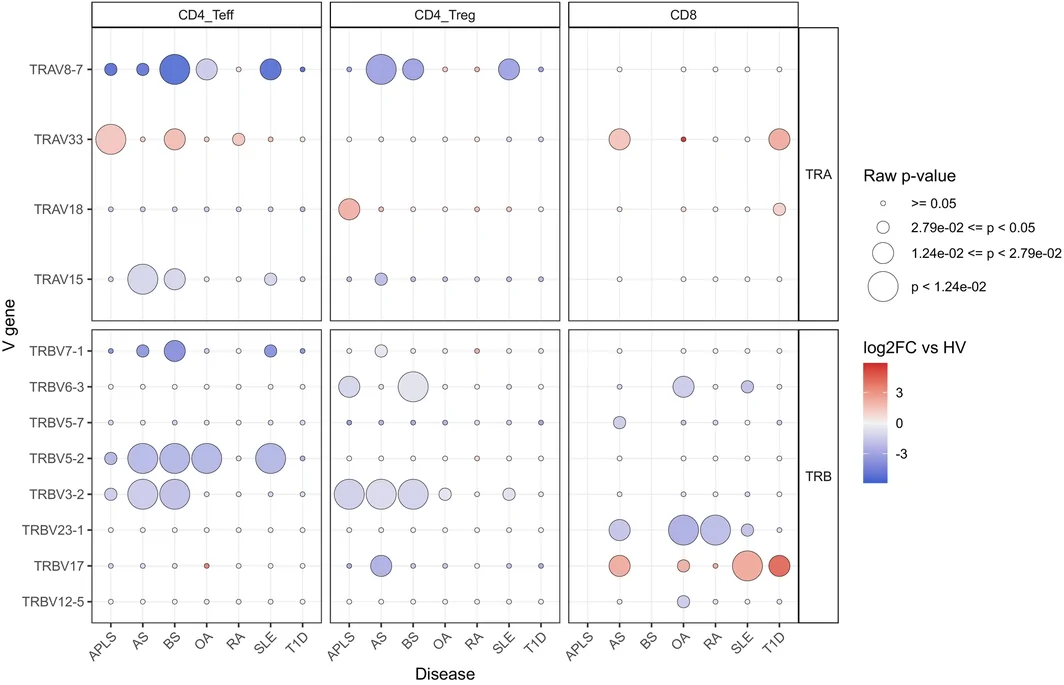
V genes usage in autoimmune diseases, Transimmunom trial (NCT02466217). Per‐sample V‐gene usage frequency (gene level, allele‐unresolved) for TRA and TRB, shown as log2 fold change (log2FC) between healthy volunteers (HV) and each disease group. Dot color represents the direction and magnitude of fold change; dot size represents statistical significance (Mann‐Whitney U test, *p* < 0.05). APLS, antiphospholipid syndrome; AS, ankylosing spondylitis; BS, Behçet syndrome; OA, osteoarthritis; RA, rheumatoid arthritis; SLE, systemic lupus erythematosus; T1D, type‐1 diabetes.

Skewed V‐gene usage has been reported across multiple autoimmune settings. In SLE and RA, significant differences in V‐gene usage, J‐gene usage, and V‐J pairing have been observed between patients and healthy controls, with V‐gene usage alone showing strong discriminatory performance between disease and control repertoires with an AUC of 0.99 [122]. In T1D, antigen‐specific T cells show epitope‐dependent V‐J constraints: IGRP‐reactive CD8+ T cells display near‐universal TRAJ53 usage shared across patients [133], whereas GAD65‐specific CD4+ T cells show broadly diverse TRBV usage with no cross‐patient convergence [134]. Similar patterns of biased TRBV usage have been described in autoimmune thyroid diseases, with Graves' disease and Hashimoto's thyroiditis each showing preferential usage of partially overlapping but disease‐specific TRBV genes, suggesting that V‐gene skewing may reflect distinct antigenic targets even within related autoimmune conditions [135]. In Crohn's disease, altered gene usage has been observed in intestinal repertoires, with several TRAV, TRAJ, and TRBV genes showing significantly decreased usage in patients compared with controls [126]. Consistent with the patterns described above, an exploratory analysis of an in‐house dataset from the Transimmunom trial (NCT02466217, [136]), a multi‐autoimmune and inflammatory disease cohort of patients, similarly revealed preferential V gene usage per disease (TRA and TRB) compared to healthy volunteers across several conditions including SLE, RA, and T1D, as well as some genes shared between diseases (Figure 3). Together, these findings suggest that autoimmune repertoires may be enriched for specific V/J gene combinations, potentially reflecting genetic bias in certain populations, including HLA and likely preferential recognition of disease‐relevant peptide‐MHC complexes.

Alterations in CDR3 length and amino acid composition are more context‐dependent than V/J gene skewing. Because CDR3 loops are subject to thymic selection constraints that disfavor excessively long or physicochemically extreme sequences, autoimmune repertoires do not necessarily show global disruption of CDR3 architecture. Some studies report near‐normal global CDR3 length distributions in autoimmune disease; for instance, CDR3 length distributions are comparable between SLE patients and healthy controls [137], while others identify disease‐specific shifts: in T1D, CDR3β are consistently shorter than in healthy donors across multiple CD4+ T cell subsets, introduced by increased deletions and reduced insertions during VDJ rearrangement, suggesting that early repertoire generation itself may be abnormal [138]. Changes in amino acid composition represent an additional layer of remodeling: hydrophobic residues at specific CDR3/beta positions promote self‐reactive T cell development in T1D‐associated MHC contexts in mice [139], and in SLE, characteristic CDR3 amino acid patterns differ across T cell subsets, with hydrophobic residues enriched in regulatory T cells and acidic residues in Th1 cells, and this compositional shift correlates with disease activity [140].

In HLA‐B27‐associated axSpA, independent repertoire studies identified convergent CD8^+^ TCRs characterized by preferential TRBV9–TRBJ2‐3 usage and related CDR3β motifs, with disease‐associated clonotypes enriched in synovial fluid [65, 66, 67, 68]. Subsequent paired‐chain analyses refined this interpretation by showing that antigen recognition cannot be attributed to TRBV9 usage alone [69], as TCRs cross‐reactive for self‐antigens and for a peptide shared by several enteric bacteria (YeiH‐reactive TCRs) can use alternative TRBV genes while retaining a disease‐associated CDR3β motif. These observations suggest that specificity may instead depend on structural features distributed across the complete αβ TCR. Thus, apparent V‐gene biases in repertoire data may represent accessible markers of a deeper structural convergence rather than the molecular determinant of antigen specificity itself.

Overall, autoimmune repertoire composition shows selective biases in V/J gene usage, V‐J pairing, and, in some contexts, CDR3 length and physicochemical properties, which may constitute sequence‐level footprints of preferential structural configurations rather than direct determinants of antigen specificity.

### 3.3 Antigen‐Recognition Landscapes

Antigen‐driven selection can arise through the intrinsic cross‐reactivity of the TCR. Because the potential peptide‐MHC universe greatly exceeds the size of the TCR repertoire, individual clonotypes recognize more than one ligand [103]. Microbial mimicry is the case in which a pathogen‐derived peptide‐MHC and a self‐peptide‐MHC activate the same TCR, creating a plausible route from infection to autoreactivity [78, 141]. Several examples highlight the link between such pathogen‐reactivities and various AD.

Hiemstra et al. showed that GAD65‐reactive CD4+ T‐cell clones from a prediabetic patient with stiff‐person syndrome cross‐recognized a peptide from the human cytomegalovirus (CMV) major DNA‐binding protein. The viral ligand was identified through HLA‐DR3‐binding peptide‐library screening and was naturally processed by dendritic cells, establishing that it was accessible to the relevant T cells rather than merely a computationally plausible ligand [142]. Sethi et al. resolved structures of an MBP‐reactive TCR (Hy.1B11) derived from an MS patient with the self‐peptide and two pathogen‐derived peptides. A focused CDR3α interaction dominated the recognition: residue F95 contributed most peptide contacts across all three complexes, while E98 provided a critical MHC contact [143]. Harkiolaki et al. further showed that a bacterial peptide sharing a structural recognition hotspot with MBP could activate the same TCR and induce MS‐like disease in humanized mice [144]. Molecular‐dynamics simulations of Hy.1B11 subsequently supported this structural model, identifying a similar bridging interaction pattern across the self and microbial peptide‐MHC complexes and reinforcing the importance of the CDR3α E98‐MHC interaction [145]. Similarly, in HLA‐B27‐associated axSpA, the bacterial YeiH peptide and structurally related self‐peptides presented by HLA‐B*27:05 can be recognized by the same disease‐associated TCRs, involving particular motifs on the CDR3β and the CDR1 of TRAV21, providing additional evidence for molecular mimicry based on shared structural features of microbial and self pMHC ligands in AD [68, 69]. In their study, Paley et al. showed that YeiH‐specific cells display phenotypic and transcriptional features associated with intestinal differentiation, further supporting a possible link between mucosal exposure to microbial antigens and the emergence of cross‐reactive T‐cell responses.

Cross‐reactivity does not require extensive overall TCR sequence identity. This distinction matters for prediction. Cross‐reactive ligand sets may retain a conserved TCR‐facing recognition motif despite substantial diversity elsewhere in the peptide sequence [146]. Structure‐based modeling can therefore complement sequence‐ and motif‐based specificity inference: Borrman et al. used TCR–pMHC modeling and energetic scoring to rank known cross‐reactive ligands from candidate pools containing more than 10^5^ peptides [147]. Whether such cross‐reactive clones become clinically consequential will depend on the timing and anatomical context of antigen exposure.

### 3.4 Spatio‐Temporal Dynamics of Autoimmune Repertoires

The autoimmune T cell repertoire is not a single entity but a spatially distributed, temporally evolving population. Peripheral blood samples a minority of disease‐associated clonotypes: in RA, the dominant T cell clones in inflamed synovium are largely absent from blood, with cross‐tissue overlap as low as 4%, while the same clones are consistently shared across multiple joints in the same patient [45, 148]. This pattern extends beyond classical autoimmunity; systematic multi‐tissue TCR sequencing in graft‐versus‐host disease, an alloimmune model of tissue‐targeted inflammation, found that blood and spleen captured only a limited fraction of the tissue repertoire, with anatomic proximity between tissues being a stronger determinant of repertoire composition than disease status [149]. In lymphoid organs, disease‐associated clonal expansions can precede and persist through clinical onset, suggesting that draining lymph nodes harbor repertoire dynamics that blood sampling would miss entirely [150].

Beyond spatial compartmentalization, the repertoire is also temporally dynamic. Early RA synovium is dominated by a small number of highly expanded private clones that contract as disease becomes established, with highly expanded clones accounting for 56% of sequenced TCRs in early disease versus only 9.8% in established RA [45]. In juvenile idiopathic arthritis, dominant regulatory and conventional T cell clones were similarly found to be compartmentalized and persistent, supporting the concept of antigen‐skewed repertoire architecture [151]. Similarly, in systemic sclerosis and multiple sclerosis, antigen‐driven clones have been shown to persist at high frequency over periods of years, suggesting that tissue‐resident and circulating autoreactive populations are maintained by ongoing antigenic stimulation [152, 153]. The relevance of these dynamics extends to other immune‐mediated conditions: cardiovascular diseases are increasingly recognized as having an immune component, and consistent with this, Le Gouge et al. identified a peripheral blood TCR signature associated with 1‐year cardiac functional outcome after myocardial infarction, despite the absence of major global differences in repertoire diversity or gene usage; the antigenic specificity and mechanisms underlying this association remain unresolved [154, 155].

Understanding disease pathogenesis therefore requires sampling beyond blood, at tissue sites proximal to inflammation and at time points that reflect the relevant phase of disease.

### 3.5 Public Clonotypes and Repertoire Sharing

Public clonotypes, defined as identical or highly similar TCR sequences detected across individuals, represent a recurrent but quantitatively limited feature of TCR repertoire architecture. Exact overlap between individuals is typically low, often representing less than 1% of TCR sequences even when millions of clonotypes are analyzed, reflecting the enormous diversity of V(D)J recombination and the predominance of private clonotypes [156, 157]. Nevertheless, this small shared fraction is biologically and analytically important. They are enriched among sequences with high generation probability, abundant or persistent clones, and receptors shaped by convergent recombination or shared selection pressures, rather than necessarily reflecting shared antigen exposure [158]. In unrelated individuals, Shugay et al. reported extensive sharing at the amino acid level when repertoires were deeply sequenced, with approximately 72,000 amino acid‐identical CDR3 variants shared between pairs at maximal sequencing depth (~10^6^ unique sequences per donor), compared to only approximately 6000 at the nucleotide level for the same pairs [159]. This striking divergence indicates that identical amino acid sequences frequently arise through distinct nucleotide rearrangements, supporting the concept of convergent recombination, whereby the same CDR3 amino acid sequence can be generated through multiple independent recombination events [160].

Twin studies further illustrate the balance between shared genetic constraints and individualized immune history. Zvyagin et al. found that V gene segment usage for both TCRβ and TCRα chains is more similar within identical twin pairs than between unrelated individuals, including among out‐of‐frame sequences, suggesting that genetic background can influence recombination before thymic selection [161]. J gene usage showed weaker genetic determination at the recombination stage, whereas Jα usage became more similar within twins after thymic selection, consistent with MHC‐dependent shaping of the TCRα chain. However, even genetically identical individuals retain highly personalized immune responses: in yellow fever vaccination studies, vaccine‐responding TCR repertoires in identical twins showed convergent features but limited exact sharing, with each individual generating a largely private set of hundreds to thousands of reactive clonotypes [162].

These observations have important implications for autoimmune disease. Public autoimmune‐associated clonotypes may exist, particularly when patients share HLA alleles or disease‐relevant antigens, yet their detection is complicated by sparse sampling, private clonal expansion, and the low overall overlap between individuals. Detecting such clonotypes is further complicated by the need to interpret repertoire sharing in light of clone frequency, sampling depth, and the long tail of rare private sequences [159]. Thus, public clonotypes represent the detectable fraction of a broader antigen‐driven landscape, while much of the disease‐relevant signal may remain hidden among low‐frequency, private, or structurally similar clonotypes, a gap that emerging methods for clonotype clustering, motif detection, and antigen prediction may begin to address.

### 3.6 Evidence of Antigen‐Driven Selection and Biomarker Potential

Antigen‐driven selection can leave detectable signatures in the TCR repertoire, yet these signatures are often subtle, sparse, and distributed across only a small fraction of the total repertoire. In practice, disease‐associated clonotypes may represent only a minor subset of all observed sequences, making their detection a “needle‐in‐a‐haystack” problem [163, 164]. This issue is particularly relevant in autoimmune disease, where the relevant T‐cell response may be private, tissue‐enriched, or only weakly expanded in peripheral blood. As a result, single repertoire features are rarely sufficient for robust diagnosis or stratification, and multifeature or ensemble approaches that integrate clonal expansion, sequence similarity, V/J gene usage, CDR3 motifs, physicochemical properties, and repertoire‐level summary statistics are increasingly favored [165, 166, 167, 168].

Despite these limitations, repertoire features can carry clinically informative signals. In a landmark study of viral exposure, Emerson et al. showed that TCR repertoire signatures could identify cytomegalovirus exposure history and reveal HLA‐mediated effects on repertoire composition, supporting the broader concept that antigenic history is encoded in circulating TCR repertoires [169]. Although infection‐associated signals are often stronger and more public than autoimmune signals, this work provides a conceptual framework for using repertoire‐level information to infer immune exposure, disease state, or clinical trajectory. Building on this concept, machine learning approaches are used to extract disease‐specific TCR signatures in autoimmune contexts. Liu et al. identified 198 SLE‐associated TCRβ clonotypes from 877 patients, achieving near‐perfect classification accuracy, while Rawat et al. used deep learning to identify T1D‐associated CDR3 motifs enriched in both peripheral blood and pancreas‐draining lymph nodes, offering a potential basis for TCR‐based diagnostics [122, 128].

Autoimmune repertoire formation is shaped not only by peripheral antigen exposure but also by preselection recombination biases and thymic selection, which determine which potentially autoreactive clones enter the mature T‐cell pool. Experimental and conceptual work has shown that the mature TCR repertoire is partitioned by selection constraints and MHC recognition, with CDR3 sequence features influencing which receptors survive selection [170]. This thymic component may be particularly relevant for autoimmune disease. Recent work further suggests that thymic selection may differ by sex, with female repertoires showing enrichment of TCR specificities associated with autoimmunity, offering a potential mechanism for sex‐biased autoimmune susceptibility [171].

Overall, repertoire‐based biomarkers hold substantial promise for autoimmune disease. The most robust approaches will need to combine biological knowledge of antigen‐driven selection with statistically rigorous analysis, longitudinal sampling, tissue‐aware study designs, and interpretable machine‐learning models.

## 4 Major Limitations

Despite major progress in adaptive immune receptor repertoire (AIRR) sequencing and analysis, several limitations still constrain the interpretation of TCR repertoire data in autoimmune diseases. These limitations are both technical and biological. In practice, the study of autoimmune repertoires is affected by methodological variability, incomplete sampling of the relevant immune compartments, limited access to antigen specificity, and cohort heterogeneity. Together, these issues help explain why many findings remain difficult to compare across studies and why translation to clinically useful biomarkers is still not achieved [121, 168, 172].

### 4.1 Technical Bias

A first limitation in the field is the substantial variability introduced at every step of the experimental and computational workflow [121, 173, 174]. TCR repertoire datasets can differ according to sample handling, nucleic acid source, amplification strategy, sequencing platform, sequencing depth, and downstream bioinformatics processing [173, 174]. As a result, differences observed between studies may reflect methodological heterogeneity as much as disease‐related biology [174, 175]. TCR repertoires are influenced by the type of starting material, including whole blood, PBMCs, sorted T‐cells, or tissue [121, 173]. The amount of input material, the quality of the sample, cryopreservation procedures, and cell‐sorting strategies can all affect which clonotypes are captured and with what abundance [173, 174, 175]. The choice between RNA‐ and DNA‐based protocols also has important consequences. DNA‐based approaches may better reflect clonal frequency at the cellular level, whereas RNA‐based approaches can provide higher sensitivity for expressed TCRs, especially in low‐input samples, at the cost of abundance estimates being influenced by transcriptional activity [121, 173, 174].

TCR repertoires can also be affected by amplification and sequencing biases [173, 174]. Multiplex PCR, 5′ RACE‐based approaches, and other library preparation strategies differ in their sensitivity, V/J coverage, susceptibility to primer bias, and ability to recover low‐frequency clonotypes [173, 174]. These differences are particularly problematic when comparing studies that report shifts in V/J usage, CDR3 length distributions, or clonal expansions because such features can be strongly influenced by technical choices [174, 175]. In autoimmune diseases, where disease‐associated signals are often subtle and can involve a minority of the total repertoire, even small technical distortions may substantially affect biological interpretation [121, 168].

Analytical variability further complicates interpretation [121, 168, 173]. Repertoire metrics depend on the definition of clonotypes, the treatment of sequencing errors, the handling of singletons, the normalization strategy, and the choice of diversity indices [168, 176, 177]. Different software pipelines may generate different estimates of clonotype abundance, diversity, convergence, or sharing even when applied to the same raw data [173, 176, 178]. Likewise, the annotation of V, D, and J genes and the treatment of ambiguous assignments can alter the apparent structure of the repertoire [178, 179, 180]. This issue becomes especially important when attempting to compare public datasets or build multi‐cohort studies including multi‐disease comparisons [168, 172, 180].

The field has made important efforts toward standardization, notably through community guidelines, benchmarking studies, and biological controls [168, 172, 175, 180]. However, full harmonization remains incomplete, and many published studies still rely on workflows that differ substantially in acquisition and analysis [121, 174, 175]. This technical variability is especially problematic in autoimmunity because repertoire alterations are often modest relative to the broader background of immune history, age, infections, and treatment exposure [121, 168].

### 4.2 Biological Sampling Bias

Even when technical variability is minimized, repertoire studies remain constrained by a more fundamental problem: only a very small fraction of the total T‐cell repertoire is actually sampled [121, 181, 182]. In autoimmune diseases, this limitation is critical because the most relevant clonotypes may be rare, spatially restricted, or transiently expanded [123, 151]. The sequenced repertoire is therefore not a complete representation of the immune response, but rather a snapshot shaped by sampling depth, input material, biological compartment, and timing.

This problem is first quantitative. Only a subset of all circulating or tissue‐resident T cells is collected, and only a subset of those cells is sequenced [181, 182]. Rare clonotypes are particularly vulnerable to under‐sampling, which can lead to unstable estimates of diversity, clonal structure, and repertoire overlap [181, 182]. This is already an issue in large blood samples and becomes even more severe in small biopsies, sorted subpopulations, and single‐cell experiments [121, 183]. In such settings, the absence of a clonotype from the dataset cannot be interpreted as evidence of its true biological absence [121, 181, 182].

However, the limitation is not only quantitative; it is also anatomical and temporal. In many autoimmune disorders, blood is the most accessible compartment and therefore the most frequently studied. However, peripheral blood may incompletely reflect the disease‐relevant immune processes occurring in tissues and draining lymph nodes [123, 151]. Tissue‐restricted clones may be diluted in blood, whereas circulating clones may not be the principal drivers of local pathology [123, 151]. This disconnection is especially important in organ‐specific autoimmune diseases, but it could also apply to systemic conditions in which pathogenic clones may redistribute across compartments over time [123, 151].

Temporal sampling adds another layer of uncertainty. Autoimmune diseases are dynamic, with phases of flare, remission, chronic low‐grade inflammation, and treatment‐induced remodeling. A clonotype that is highly relevant at one stage of disease may be undetectable at another [123, 151]. Lack of patient stratification might therefore risk confounding disease‐relevant variation with transient immune fluctuations unrelated to the pathogenic process itself [121, 180]. This issue is magnified by the fact that TCR repertoires are shaped by many exposures other than autoimmunity, including infections, vaccination, aging, and turnover [169, 184, 185]. Overall, biological sampling bias means that autoimmune TCR repertoire datasets should be interpreted as partial, compartment‐dependent, and time‐dependent views of disease‐associated immunity rather than exhaustive maps of pathogenic T‐cell responses.

### 4.3 The Specificity Gap

A central limitation of TCR repertoire analysis is that sequence information alone does not directly provide antigen specificity or functional meaning [121, 186]. Although repertoire sequencing can describe clonal expansion, diversity, sharing, convergence, and sequence architecture at a large scale, it does not by itself identify the targeted antigens, the relevant peptide‐MHC context, the strength of interaction, or the pathogenic relevance of individual clonotypes [186, 187, 188]. This gap between sequence and function remains one of the main obstacles to mechanistic interpretation in autoimmune diseases [121, 189].

This limitation distinguishes repertoire analysis from more directly interpretable approaches. In transcriptomic analyses, for example, measured features can often be linked to annotated genes, pathways, and cellular states. In contrast, a TCR sequence is informative in a more indirect way: it encodes antigen recognition, yet the peptide‐MHC ligand remains unknown for the vast majority of TCRs [121, 186]. Consequently, repertoire analysis often relies on surrogate readouts and computational approximations, including clonal expansion, motif enrichment, sequence‐based distances, network‐based clustering, embedding or vector representations, protein‐language‐model representations, and structure‐informed modeling to approximate antigen‐driven biology. Similarity and clustering‐based methods, such as TCRdist, GLIPH/GLIPH2, TCRex, TCRMatch, GIANA, iSMART, and clusTCR, group TCRs according to CDR3 sequence similarity, biochemical features, shared motifs, or learned distances, with the aim of identifying convergent receptors that may recognize related antigens [190, 191, 192, 193, 194]. Supervised binding predictors, including ERGO, NetTCR, ImRex, TITAN, DLpTCR, DeepTCR, and related models, instead learn associations between TCR sequence features, peptide sequence, HLA context, and antigen specificity or disease status [195]. More recent approaches use transformer‐based architectures and protein language models to generate contextual sequence embeddings, capturing constraints that are not easily represented by sequence distance metrics [196, 197, 198]. In parallel, multimodal single‐cell models such as mvTCR and CoNGA [199, 200] integrate TCR identity with transcriptomic or surface‐protein phenotypes, while structure‐aware methods attempt to model TCR or TCR‐peptide‐MHC geometry. These structural approaches build on the precedent set by AlphaFold for protein structure prediction and now include TCR‐specific or immune‐receptor‐focused tools such as TCRmodel/TCRmodel2, TCRBuilder2, ImmuneBuilder, TFold, and AlphaFold‐derived strategies [201, 202, 203, 204]. Despite these advances, all of these methods remain constrained by sparse and biased training data, uneven HLA and epitope coverage, uncertain negative examples, limited paired αβ information, and the small number of experimentally validated TCR‐peptide‐MHC structures relative to the enormous diversity of the repertoire. These surrogate approaches are useful, but they remain proxies. TCRs with similar sequences do not necessarily recognize the same antigen, and TCRs with distinct sequences may converge functionally on related peptide‐MHC targets [187, 192, 205]. In addition, TCR recognition is not strictly one receptor to one antigen. Cross‐reactivity and polyspecificity are intrinsic features of T‐cell recognition, and many receptors likely engage a spectrum of ligands with different affinities and functional consequences [103, 104, 206, 207]. This has major implications for autoimmunity, where disease‐relevant clones may be shaped by self‐antigens, environmental triggers, microbial mimicry and bystander activation [189, 206, 208].

Current databases of TCR specificities provide valuable resources, but they remain limited in size and coverage [205, 209, 210, 211]. Known TCR‐antigen pairs cover only a very small fraction of the total sequence space observed in repertoire studies [121, 186, 205]. The available data are also unevenly distributed across HLA contexts, pathogens, epitopes, and experimental systems [186, 187, 205, 212]. This sparsity limits the performance and generalizability of computational models trained to predict specificity, especially for unseen epitopes or poorly represented HLA backgrounds [164, 187, 212, 213]. The absence of robust negative datasets is also a problem for training generalizable antigen‐specificity predictive models, since the lack of demonstrated binding does not establish true nonspecificity [186, 212, 213]. In autoimmune contexts, where rare and poorly characterized self‐reactive populations are precisely the populations of interest, these limitations become especially severe [189, 214, 215].

Experimental approaches can help bridge the specificity gap, including peptide‐MHC multimer staining, antigen stimulation assays, and single‐cell multimodal profiling [62, 215, 216, 217, 218]. However, these methods are generally lower throughput than bulk repertoire sequencing and are often restricted to predefined candidate antigens or HLA contexts [189, 216, 217]. They also do not fully resolve the distinction between antigen recognition, activation state, and pathogenicity. A clonotype may bind a given peptide‐MHC complex without being pathogenic, or may contribute to pathology only under inflammatory conditions [214, 215].

### 4.4 Cohort Heterogeneity

Autoimmune diseases are themselves clinically heterogeneous, with differences in disease duration, activity, organ involvement, flare status, treatment exposure, and immune history. These variables can strongly influence repertoire composition and may obscure disease‐specific signals [168, 183, 219]. This issue is further amplified by HLA diversity, which is central to T‐cell antigen recognition [10, 220, 221]. Because TCR selection and peptide recognition are constrained by peptide‐HLA presentation, patients with different HLA backgrounds may mount distinct autoreactive T‐cell responses even within the same diagnostic category. Conversely, shared HLA risk alleles may favor partially convergent repertoire features, making HLA stratification important when interpreting V/J gene usage, CDR3 motifs, clonotype sharing, or disease‐associated signatures [161, 169, 221, 222]. This convergence depends on the degree of HLA overlap between individuals. Patients sharing little to no HLA background may present distinct sets of epitopes and mount different autoreactive T‐cell responses. On the other hand, shared HLA alleles can generate overlapping peptide‐HLA landscapes and induce common TCR responses. Population structure is therefore also important, since HLA allele frequencies, genetic risk architecture, environmental exposures, and infectious history differ across ancestry groups and geographic settings. As a result, repertoire features identified in one cohort may not generalize to another, particularly when cohorts are small, poorly matched, or insufficiently stratified.

Additionally, disease‐relevant responses may be dominated by private or poorly shared clonotypes, reducing portability across individuals, cohorts, and sequencing platforms. In addition, most TCR sequencing experiments sample only a fraction of the full repertoire, so the apparent absence of a clone may reflect undersampling rather than true biological absence [182]. This limitation is particularly important in relatively “cold” autoimmune settings, or in compartments where antigen‐driven expansion is modest and disease‐associated clones are diluted by abundant background T cells. These challenges complicate the distinction between true antigen‐driven selection and apparent enrichment caused by sampling depth, clonal abundance, or generation probability.

### 4.5 Limited Biological Interpretability of Total TCR Repertoires

As described in the previous sections, most studies of autoimmune diseases have relied on bulk sequencing of total T‐cell repertoires obtained from whole peripheral blood, implicitly assuming that the resulting repertoire constitutes a coherent biological entity. However, this assumption deserves reconsideration. Rather than representing a single biological system, a bulk repertoire is the superposition of multiple repertoire compartments originating from distinct T‐cell populations, each characterized by its own developmental history, selection mechanisms, antigenic exposures, homeostatic constraints and clonal dynamics. Studies performed on purified T‐cell populations have consistently demonstrated that repertoire architecture is intrinsically linked to cellular differentiation. Distinct repertoire organizations have been described not only between conventional naïve and memory T cells [185, 223, 224], but also within the regulatory T‐cell lineage itself, where naïve and activated/memory Tregs display markedly different clonal structures and tissue distribution, reflecting their distinct developmental trajectories and biological functions [16, 225]. In disease, naïve and activated/memory subsets display distinct repertoire dynamics and biological functions [226, 227, 228]. These findings illustrate how biologically relevant signals may be diluted and hence become undetectable when broader lymphocyte populations are analyzed as a whole.

Importantly, the composition of peripheral T‐cell populations is itself highly dynamic throughout life. Progressive thymic involution reduces the production of naïve T cells, while cumulative antigenic exposure progressively drives the expansion and stabilization of memory clones, profoundly reshaping repertoire architecture with age [223, 224, 229]. Persistent viral infections, particularly CMV, further remodel the repertoire by promoting large antigen‐driven clonal expansions that may dominate substantial fractions of the circulating T‐cell pool [169]. Vaccination history, environmental exposures, host genetics, immunomodulatory therapies, and disease activity further modify both the relative abundance of immune cell subsets and their intrinsic repertoire properties.

Consequently, any repertoire metric calculated on the total repertoire reflects a composite signal whose biological interpretation is inherently ambiguous. Changes in diversity, clonality, V/J gene usage, or public clone frequency may arise either from disease‐associated antigen‐driven responses or simply from shifts in the relative abundance of cellular subsets (as depicted in Figure 3 for V gene usage). These proportions themselves are influenced by numerous variables, including age, chronic viral infections, vaccination history, immune senescence, genetic background, ongoing treatments, and disease activity, making it difficult to disentangle true repertoire remodeling from changes in cellular composition. This complexity likely contributes to the limited reproducibility of many repertoire‐derived biomarkers across autoimmune diseases and independent cohorts.

More fundamentally, repertoire analyses often compare distributions that are likely not biologically equivalent. The naïve compartment predominantly reflects thymic output and central tolerance, whereas memory subsets capture the cumulative history of antigenic encounters; regulatory T cells are shaped by distinct thymic and peripheral selection pressures [227, 230, 231, 232], and terminally differentiated populations are frequently dominated by large antigen‐driven clonal expansions [169, 185]. Collapsing these compartments into a single repertoire therefore combines multiple biological processes operating at different temporal and mechanistic scales into a single statistical object. Such averaging inevitably dilutes disease‐specific signals while simultaneously introducing confounding effects that cannot readily be resolved by increasing cohort size or sequencing depth alone.

Future studies should move beyond the concept of the “total repertoire” and instead aim to characterize biologically meaningful repertoire compartments. Importantly, this does not imply replacing bulk repertoire sequencing with single‐cell technologies. Rather, these approaches provide complementary information. Bulk sequencing of purified T‐cell populations remains the most powerful strategy for exhaustively characterizing repertoire diversity, clonal architecture, and low‐frequency clonotypes within a defined cellular subset, owing to its substantially greater sequencing depth and sensitivity. Conversely, single‐cell immune repertoire profiling combined with transcriptomic and phenotypic characterization enables clonotypes to be assigned to specific cellular identities, differentiation states, and functional programs [200, 233, 234]. However, depending on the type of sample, blood or tissue, the throughput needs to be adapted or cell subsets to be sorted prior to single‐cell so that clonal diversity is accurately measured. Interpreting repertoire architecture within defined cellular contexts is likely to substantially improve both the mechanistic understanding of autoimmune diseases and the reproducibility of repertoire‐based biomarkers.

## 5 Future Directions

Future directions should aim to move autoimmune TCR repertoire analysis from descriptive sequence catalogues to biologically grounded and clinically interpretable models. Longitudinal and multi‐compartment studies integrating blood and tissue repertoires with single‐cell transcriptomics, paired αβ TCR sequencing, HLA background, spatial context, and B‐cell/autoantibody data will help connect disease‐associated clonotypes to cellular state, tissue localization, and disease evolution. Large‐scale multimodal reference atlases may further identify the cellular compartments carrying the most informative disease‐associated repertoire signals.

However, while tissue‐derived and single‐cell datasets are invaluable for biological discovery, they are unlikely to become routine diagnostic tools. Tissue sampling remains invasive, and current single‐cell technologies remain expensive, technically demanding, and difficult to standardize for large‐scale clinical implementation. Peripheral blood will therefore continue to represent the most practical and scalable source of immune repertoire information for diagnosis, prognosis, and treatment monitoring.

Consequently, one of the major challenges for the coming years will be translating the biological knowledge generated by high‐resolution studies into clinically deployable assays. Rather than replacing bulk sequencing, single‐cell technologies should serve as the reference from which simpler approaches can be developed. Computational deconvolution methods, repertoire analyses restricted to selected cell subsets, multimodal machine learning models, and targeted sequencing strategies may ultimately provide affordable, standardized, and high‐throughput solutions suitable for clinical laboratories.

Finally, closing the antigen‐specificity gap remains central to this effort. Broader and better curated TCR‐peptide‐MHC databases are needed, but computational prediction will need to be complemented by functional validation, including peptide‐MHC binding, antigen‐stimulation assays, T‐cell activation assays, and single‐cell multimodal profiling. Recent higher‐throughput wet‐lab specificity assays, including display‐based peptide‐recognition profiling, pooled antigen‐presentation or activation screens, scalable TCR‐library screening, and single‐cell multimodal readouts [235, 236, 237, 238, 239, 240, 241], will be critical to bridging this gap. Ultimately, clinically useful repertoire biomarkers will have to be not only predictive, but also interpretable, antigen‐linked, and functionally grounded, supporting diagnosis, patient stratification, monitoring of disease activity, prediction of treatment response, and the development of antigen‐specific or clone‐targeted therapies.

## 6 Concluding Remarks

**FIGURE 4:**
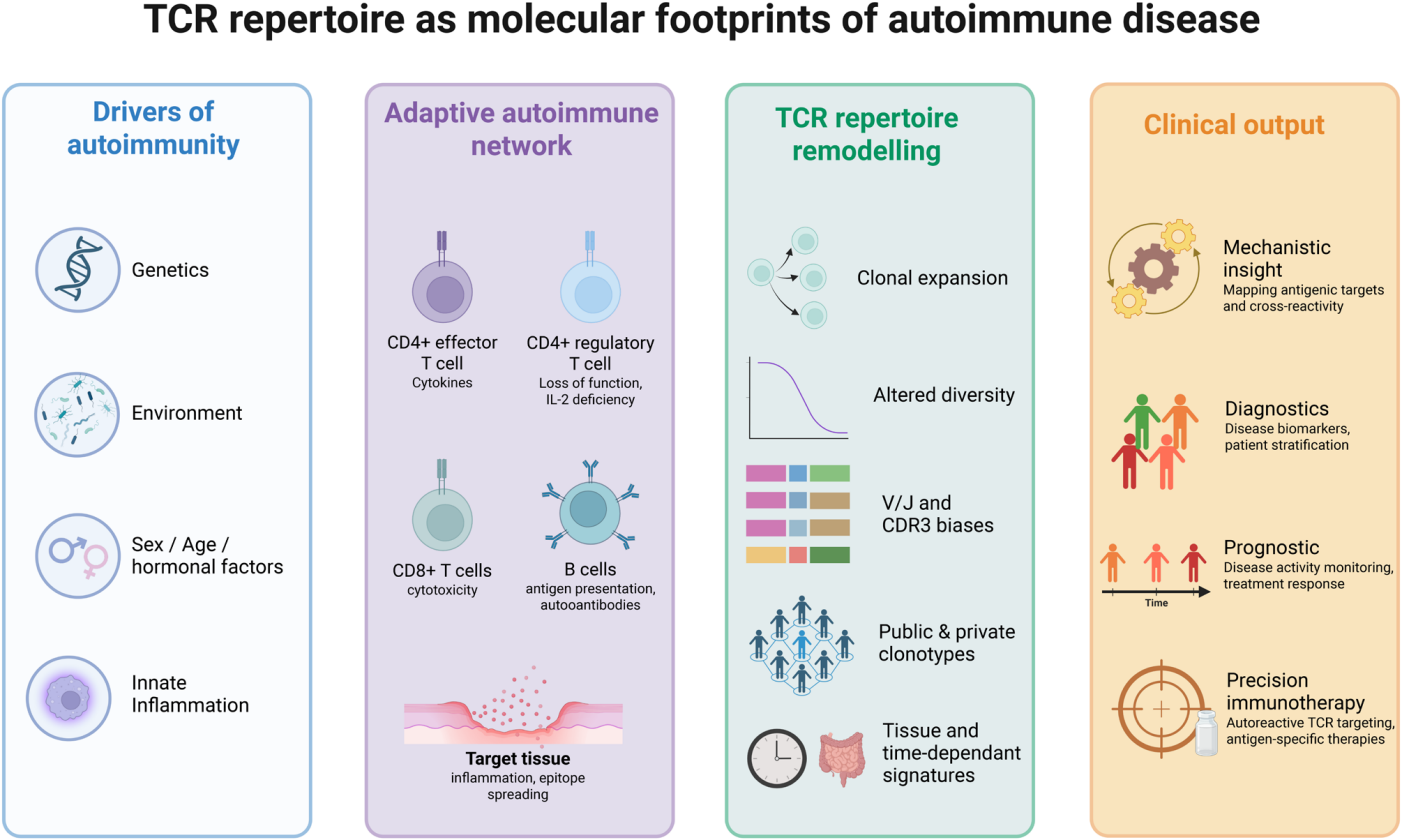
TCR Repertoire as molecular footprints of autoimmune disease. Genetic, environmental, demographic, sex, and innate inflammatory factors shape adaptive autoimmune networks and contribute to remodeling of the T‐cell receptor repertoire. Interactions among CD4+ effector T cells, CD4+ regulatory T cells, CD8+ T cells, B cells, and inflamed target tissues can drive clonal expansion, altered repertoire diversity, V/J gene and CDR3 biases, public and private clonotypes, and tissue or time‐dependent repertoire signatures. These repertoire features may provide mechanistic insight into antigenic targets and cross‐reactivity, support biomarker discovery and patient stratification, inform prognosis, and guide precision immunotherapeutic strategies.

Collectively, the studies reviewed here support a shift in how TCR repertoires are viewed in autoimmune diseases. Rather than representing a simple collection of antigen receptor sequences, TCR repertoires constitute dynamic molecular footprints of adaptive immunity that integrate the cumulative effects of genetic predisposition, environmental exposures, immune history, and ongoing tissue inflammation. When interpreted within their appropriate biological context, these footprints provide a unique systems‐level view of autoimmune responses, linking immune mechanisms to clinical phenotypes. This conceptual framework places immune repertoire profiling at the crossroads of mechanistic immunology and precision medicine, with the potential to improve disease stratification, therapeutic monitoring, and the development of antigen‐specific interventions (Figure 4).

## Funding

This work was supported by Ministère de l'Enseignement Supérieur et de la Recherche Scientifique and Institut Universitaire de France.

## Conflicts of Interest

The authors declare no conflicts of interest.

## Data Availability

Data sharing not applicable to this article as no datasets were generated or analysed during the current study.
